# Genome-wide identification and gene expression pattern of ABC transporter gene family in *Capsicum* spp.

**DOI:** 10.1371/journal.pone.0215901

**Published:** 2019-04-30

**Authors:** Carlos Lopez-Ortiz, Sudip Kumar Dutta, Purushothaman Natarajan, Yadira Peña-Garcia, Venkata Abburi, Thangasamy Saminathan, Padma Nimmakayala, Umesh K. Reddy

**Affiliations:** 1 Department of Biology, Gus R. Douglass Institute, West Virginia State University, Institute, West Virginia, United States of America; 2 ICAR RC NEH Region, Mizoram Centre, Kolasib, Mizoram, India; 3 Department of Genetic Engineering, School of Bioengineering, SRM Institute of Science and Technology, Kattankulathur, India; Birla Institute of Technology and Science, INDIA

## Abstract

ATP-binding cassette (ABC) transporter genes act as transporters for different molecules across biological membranes and are involved in a diverse range of biological processes. In this study, we performed a genome-wide identification and expression analysis of genes encoding ABC transporter proteins in three *Capsicum* species, i.e., *Capsicum annuum*, *Capsicum baccatum* and *Capsicum chinense*. *Capsicum* is a valuable horticultural crop worldwide as an important constituent of many foods while containing several medicinal compounds including capsaicin and dihydrocapsaicin. Our results identified the presence of a total of 200, 185 and 187 ABC transporter genes in *C*. *annuum*, *C*. *baccatum* and *C*. *chinense* genomes, respectively. Capsaicin and dihydrocapsaicin content were determined in green pepper fruits (16 dpa). Additionally, we conducted different bioinformatics analyses including ABC genes classification, gene chromosomal location, *Cis* elements, conserved motifs identification and gene ontology classification, as well as profile expression of selected genes. Based on phylogenetic analysis and domain organization, the *Capsicum* ABC gene family was grouped into eight subfamilies. Among them, members within the ABCG, ABCB and ABCC subfamilies were the most abundant, while ABCD and ABCE subfamilies were less abundant throughout all species. ABC members within the same subfamily showed similar motif composition. Furthermore, common *cis*-elements involved in the transcriptional regulation were also identified in the promoter regions of all *Capsicum* ABC genes. Gene expression data from RNAseq and reverse transcription-semi-quantitative PCR analysis revealed development-specific stage expression profiles in placenta tissues. It suggests that ABC transporters, specifically the ABCC and ABCG subfamilies, may be playing important roles in the transport of secondary metabolites such as capsaicin and dihydrocapsaicin to the placenta vacuoles, effecting on their content in pepper fruits. Our results provide a more comprehensive understanding of ABC transporter gene family in different *Capsicum* species while allowing the identification of important candidate genes related to capsaicin content for subsequent functional validation.

## Introduction

Pepper (*Capsicum* spp.) is a member of the Solanaceae family and is closely related to potato, tomato, eggplant, tobacco and petunia. Pepper represents an important horticultural crop worldwide not only because of its economic importance, but also due to its medicinal value. Moreover, pepper fruits have been widely used as a coloring agent, food flavoring, cosmetic and pharmaceutical ingredient, and as an ornamental product. It also has nutrimental value by containing vitamins A, B, C, and E, as well as phytochemicals such as phenolic compounds, carotenoids, and capsaicin. In addition to dietary and culinary importance, capsaicinoid compounds (capsaicin and dihydrocapsaicin) of pepper have a beneficial effect for humans, including antioxidant, anticarcinogenic, antimutagenic, antiaging, and antibacterial properties [1–4].

Capsaicinoids are strongly pungent alkaloids that accumulate in the placenta of maturing *Capsicum* fruits. Pepper genotypes exhibit a wide range of capsaicinoid accumulation as a result of both environmental and genetic variability. Recently, Nimmakayala et al. [5] determined that the major markers linked to capsaicinoid synthesis are ankyrin-like protein, the IKI3 protein family and the ATP-binding cassette (ABC) transporter family, which suggests that their activity may be involved in the pungency modulations in pepper. The ABC transporter gene family represents one of the largest gene families, with a transporter activity that is conserved and ubiquitous in all living organisms [6, 7]. Most ABC transporter proteins that have been characterized are ATP-dependent membrane-bound transporters that are able to translocate a wide range of molecules such as lipids, proteins, chemotherapeutic drugs and heavy metals via intra- and extracellular membranes [8]. In addition, some ABC proteins also act as regulators of ion channels, receptors and proteins involved in mRNA translation and ribosome biogenesis [9].

Eukaryotes feature three common arrangements of ABC transporters: full-sized transporters composed of two transmembrane domains (TMDs) and two nucleotide-binding domains (NBDs), half-sized transporters containing one TMD and one NBD, and a third type with no TMDs but two NBDs [10]. The NBD is present in all three structural types and contains many key conserved motifs: Walker A, Q-loop, Walker B, D-loop, switch H-loop, and a signature motif (LSGGQ) that is exclusively found in ABC proteins, which distinguishes ABC proteins from other ATPases [11, 12].

Plant ABC transporters are divided into eight subfamilies—A, B, C, D, E, F, G, and I—based on their protein solubility, presence of TMDs, function, and amino acid sequence [10, 13]. Although there is a ninth subfamily group, the ABCH subfamily, it has not been identified in plants. Several ABC transporters genes have been characterized in plants, however, most of them were first identified as transporters contributing to detoxification processes [14]. Subsequently, the release of genomic data and development of bioinformatics analyses have led to comprehensive research on the identification of new functions, as well as the characterization of new ABC transporter gene family in diverse plants such as *Arabidopsis*, *Oryza sativa* and *Lotus japonicus* [13, 15–17], *Zea mays* [18, 19], *Brassica rapa* [20], *Brassica napus* [21] and *Vitis vinifera* [22]. Thus, apart from just detoxification functions, the understanding of several novel roles of the ABC transporter genes have been revealed. To date, it has reported that plant ABC transporters are involved in many important physiological, growth and developmental processes. Furthermore, the transport substrates of plant ABCs are divergent and include conjugated compounds, phytohormones, primary products, lipids and lipophilic compounds [23–25].

The recent release of the genome sequence of pepper [2] has provided a platform for genome-wide analysis that allow the identification and characterization of entire gene families present in hot peppers [26–28]. For instance, it makes feasible the performance of deeper studies in the ABC transporter gene family, which remains poorly understood in *Capsicum* spp. In the current study, we report a genome-wide identification and characterization of ABC transporter genes in three *Capsicum* species (i.e., *C*. *annuum*, *C*. *baccatum* and *C*. *chinense*) including sequence alignment, phylogenetic analysis, chromosomal location and expression profile of *C*. *annuum* and *C*. *chinense*. Our results lay a foundation for further functional characterization of each ABC transporter gene among *Capsicum* species and provide useful information for better understanding the role and evolution of this gene family in higher plants.

## Materials and methods

### Plant material

*C*. *annuum* cv. CM334, *C*. *baccatum* cv. PBC81 and two varieties of *C*. *chinense* (Pimenta da neyde and Naga morich) were grown in triplicate samples in an experimental field at West Virginia State University. Fruits at 6, 16 and 25 days post-anthesis (dpa) were collected from all cultivars and stored at -80°C. Quantitative analysis of capsaicin and dihydrocapsaicin content in green pepper fruits (16 dpa) were determinate with the 1200 series HPLC system (Agilent Technologies, Santa Clara, CA) [5].

### Identification of the ABC transporter genes in pepper

To identify all members of the ABC transporter gene family in the pepper genomes, the proteomes for the three *Capsicum* species were downloaded from the pepper genome platform (PGP) (http://passport.pepper.snu.ac.kr/?t=PGENOME) [2]. A local BLASTP search was used to query the full-length amino acid sequences of ABC transporter proteins from *Arabidopsis* (https://phytozome.jgi.doe.gov/pz/portal.html) [29]. All output genes were collected and confirmed by using the software HMMER3.0 [30]. *Capsicum* genes were searched with the PF00005 ABC transporter domain, PF01061 ABC-2 transporter domain and PF00664 ABC transporter transmembrane region domain, the ABC transporter domains were confirmed using the Pfam web server (http://Pfam.sanger.ac.uk/) [31]. Genes with E-value > 1E-05 and redundant genes were excluded. Candidate genes were analyzed in the SMART database (http://smart.embl-heidelberg.de/smart/set_mode.cgi?NORMAL=1) [32] to verify the presence of the NBD and TMD domains. Genes with NBD and TMD domains were considered members of the ABC transporter family in pepper, and the coding sequences (CDS) were downloaded from the PGP database. The Jackhmmer tool (https://www.ebi.ac.uk/Tools/hmmer/search/jackhmmer) [33] was used to classify the ABC transporter gene family in subfamilies by using the UniProt reference proteome database with E-value = 0.01 for sequence matches and 0.03 for hit matches.

### Sequence alignment and phylogenetic analysis

The amino acid sequences of the *Capsicum* species and *Arabidopsis* were imported into MEGAX [34] and multiple sequence alignments were carried out using ClustalW [35] with gap-open and gap-extension penalties of 10 and 0.1, respectively. The alignment file was then used to construct a phylogenetic tree based on the neighbor-joining (NJ) method. After bootstrap analysis with 1000 replicates, the tree was displayed by using the interactive Tree Of Life platform (iTOL; http://itol.embl.de/index.shtml) [36].

### Chromosomal location, *Cis*-element analysis and identification of conserved motifs

The physical chromosome location data for each ABC transporter was downloaded from the PGP database and mapped onto the 12 chromosomes of pepper by using MapInspect. The protein size, molecular weight (MW) and theoretical isoelectric point (pI) of each ABC transporter were computed by using the proteome database and sequence analysis tools on the ExPASy Proteomics Server (http://expasy.org/) [37]. For *Cis*-element analysis, all promoter sequences (1,500 bp upstream of initiation codon “ATG”) of ABCs were extracted from the pepper genome. Then, the *cis*-regulatory elements of promoters for each gene were identified by using PLACE: A database of plant *cis*-acting regulatory DNA elements (http://www.dna.affrc.go.jp/PLACE/) [38]. Protein sequence motifs were identified by using Multiple Em for Motif Elicitation (MEME) (http://meme-suite.org/tools/meme) [39]. The analysis was performed with maximum number of motifs 10 and optimum width of motif ≥50. Discovered MEME motifs were searched in the Expasy-Prosite database with ScanProsite server (https://prosite.expasy.org/scanprosite/) [40].

### Gene ontology (GO) annotation and modeling of ABC proteins

The functional annotation of ABC transporters was performed using Blast2GO software (http://www.blast2go.com). The amino acid sequences of ABC genes were imported into Blast2GO program to execute three steps: 1) BLASTp against the NCBI non-redundant protein database, 2) mapping and retrieval of GO terms associated with the BLAST results, and 3) annotation of GO terms associated with each query to relate the sequences to known protein function.

### Identification of syntenic ABC paralogs pairs and gene synteny analysis

The syntenic ABC transporter paralogs pairs were identified by searching the gene duplication across all the species with the following criteria: 1) genes with *>*70% coverage of the alignment length; 2) genes with *>*70% identity in the aligned region; and 3) a minimum of two duplication events considered for strongly connected genes [41]. For each paralog pair, the non-synonymous substitution rate (Ka), the synonymous substitution rate (Ks) and the ω (= Ka/Ks) of paralog pairs were estimated by using KaKs_Calculator 2.0 [42]. The duplication date of paralog pairs was estimated by the formula T = Ks/2λ, assuming a clock-like rate (λ) of 6.96 synonymous substitutions per 10^−9^ years [43].

### Transcriptome sequencing of *C*. *chinense* green fruits

Green fruits (16 dpa) from two different cultivars of *C*. *chinense* were used for whole-transcriptome sequencing. Total RNA was isolated from the pooled tissues of three biological replicates for each cultivar with the Plant RNA mini spin kit (Macherey-Nagel). The quantity and quality of the total RNA were analyzed with the Agilent 2100 Bioanalyzer and Qubit 4 Fluorometer (Invitrogen), respectively. The RNA sequencing libraries were prepared by using the NEBNext Ultra II RNA Library Prep Kit according to the manufacturer's protocol. The mRNAs were enriched by using magnetic beads with Oligo (dT), then fragmented into shorter fragments with a fragmentation buffer. The first-strand cDNA was synthesized from the fragmented mRNA with a random hexamer primer. The resulting cDNAs were added to sequencing adapters, and sequencing primers were used for library amplification. The insert size of the library was analyzed with Agilent 2100 Bioanalyzer (Invitrogen), and the Qubit 4 Fluorometer (Invitrogen) was used for library quantification. The RNA sequencing library from each sample was sequenced in the Illumina NextSeq 500 platform with paired-end sequencing. The resulting image files were converted to FASTQ with 2x75-bp reads. The Illumina reads were deposited with the Sequence Reads Archive (NCBI) under the following accession number PRJNA526219.

### Analysis of *C*. *chinense* transcriptome to study ABC transporter genes

The sequencing adapters and low-quality reads (Phred score QV<30) were removed by using cutadapt (https://cutadapt.readthedocs.io/en/stable/guide.html) [44] and sickle (https://github.com/najoshi/sickle) [45] respectively. The quality-filtered reads were mapped to the *C*. *chinense* reference genome [2] by using the mem algorithm of the BWA tool [46] to generate SAM alignment. The read count table for genes from *C*. *chinense* was created for all the samples by using the SAM alignment and HTSeq R package [47]. The gene expression based on the read counts were studied by reads per kilobase per million (RPKM). The RPKM values for each gene were calculated based on the read count table, the total number of reads and gene length (kb). The ABC transporters in *C*. *chinense* (CcABCs) were identified by homology search against the CDS sequences from *C*. *annuum* by using a BLASTN algorithm (identity ≥ 98% and coverage ≥ 70%). The gene annotation of the ABC transporter genes identified from *C*. *chinense* was confirmed by using the BLASTx algorithm against the NCBI non-redundant protein database.

### Expression pattern of ABC transporters in *C*. *annuum* and *C*. *chinense*

The RNA-seq gene expression data in placenta tissues (6 dpa, 16 dpa, 25 dpa) from *C*. *annuum* cv. CM334 was retrieved from the RNA-seq data published by [2]. A BLASTN search was performed (identity ≥ 98% and coverage ≥ 70%) to identify the orthologs genes between *C*. *annuum* ABC (CaABC) and *C*. *chinense* (CcABC) transporters. The RPKM expression values for identified CaABC protein genes were extracted from the dataset and a gene expression heatmap was generated for *C*. *annuum* and *C*. *chinense* orthologs by using the ClustVis web tool (https://biit.cs.ut.ee/clustvis/) [48].

### RNA isolation and quantitative real‑time PCR (qRT‑PCR)

Total RNA was isolated from pepper fruits (6, 16 and 25 dpa) by using the Plant RNA mini spin kit (Macherey-Nagel). First-strand cDNA was synthesized with 1 μg total RNA per sample by using the Super Script First-Strand Synthesis system (Invitrogen). To identify in the three *Capsicum* genomes the orthologs of the markers previously reported by [5] for the ABC transporter family, the CDS sequences for the CA06g14430 and CA11g09150 genes were downloaded from the Sol Genomics database (https://solgenomics.net/) [49] and a BLASTN search was performed (identity ≥ 98% and coverage ≥ 70%) across the three pepper genomes. Gene-specific primers for the selected *Capsicum* ABC transporter orthologs were designed by using Primer3Plus (http://www.primer3plus.com/). The qRT-PCR analysis involved a StepOnePlus Real-Time PCR System (Applied Biosystems, Foster City, CA, USA) with a total volume of 20 μL containing 1 μL cDNA template, 2 μL forward and reverse primers (10 μM), 10 μL SYBR Green PCR Master (ROX) (Roche, Shanghai) and 7 μL sterile distilled water. For each sample, three replicates were run to compute the average Ct values. The data were analyzed by the 2−ΔΔCt method [50]. Relative gene expression was normalized against that of the endogenous control β-tubulin [51].

## Results and discussion

### Capsaicin and dihydrocapsaicin content in pepper

**Fig 1:**
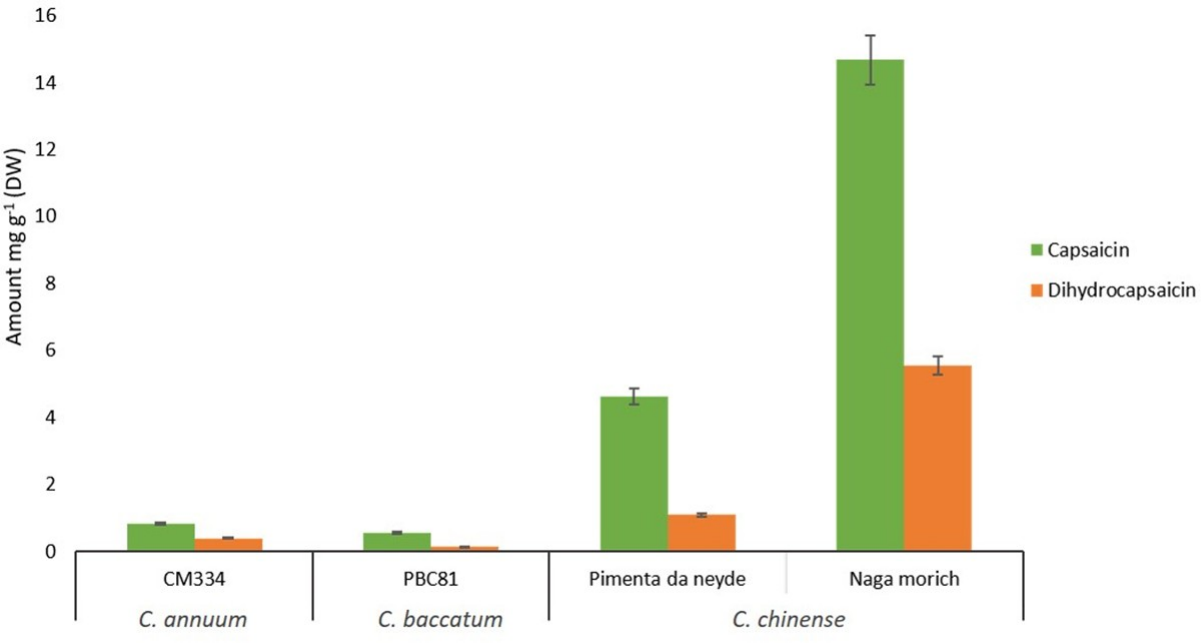
Capsaicin and dihydrocapsaicin content in pepper. Capsaicin and dihydrocapsaicin levels in pepper powder from dried green fruit (16 days post anthesis (dpa)). Values are means ± SD; n = 3.

Capsaicinoids are responsible for the hot or burning sensation of chili, pungency and flavor are the primary properties of pepper fruits [52]. About 80% to 90% of capsaicinoids in chili fruit is represented by capsaicin and dihydrocapsaicin, and their accumulation occurs over a relatively short period during the latter stages of fruit development [53]. *C*. *chinense* is one of the hottest chili peppers in the world; in general, chili species and varieties contain about 1% capsaicin, but this content can range from 2% to 4% [54]. In this study, the highest capsaicin and dihydrocapsaicin content was for *C*. *chinense* cv. Naga morich, with 14.67 mg g^-1^ and 5.54 mg g^-1^ dry weight (DW) tissue, respectively. On the other hand, 4.62 mg g^-1^ and 1.08 mg g^-1^ DW tissue were reported for *C*. *chinense* cv. Pimienta da neyde, and 0.823 mg g^-1^ and 0.393 mg g^-1^ DW tissue in *C*. *annuum* cv. CM334. The lowest value across all the species was for *C*. *baccatum*, with a content of 0.55 and 0.15 mg g^-1^ for capsaicin and dihydrocapsaicin respectively (Fig 1).

**Fig 2:**
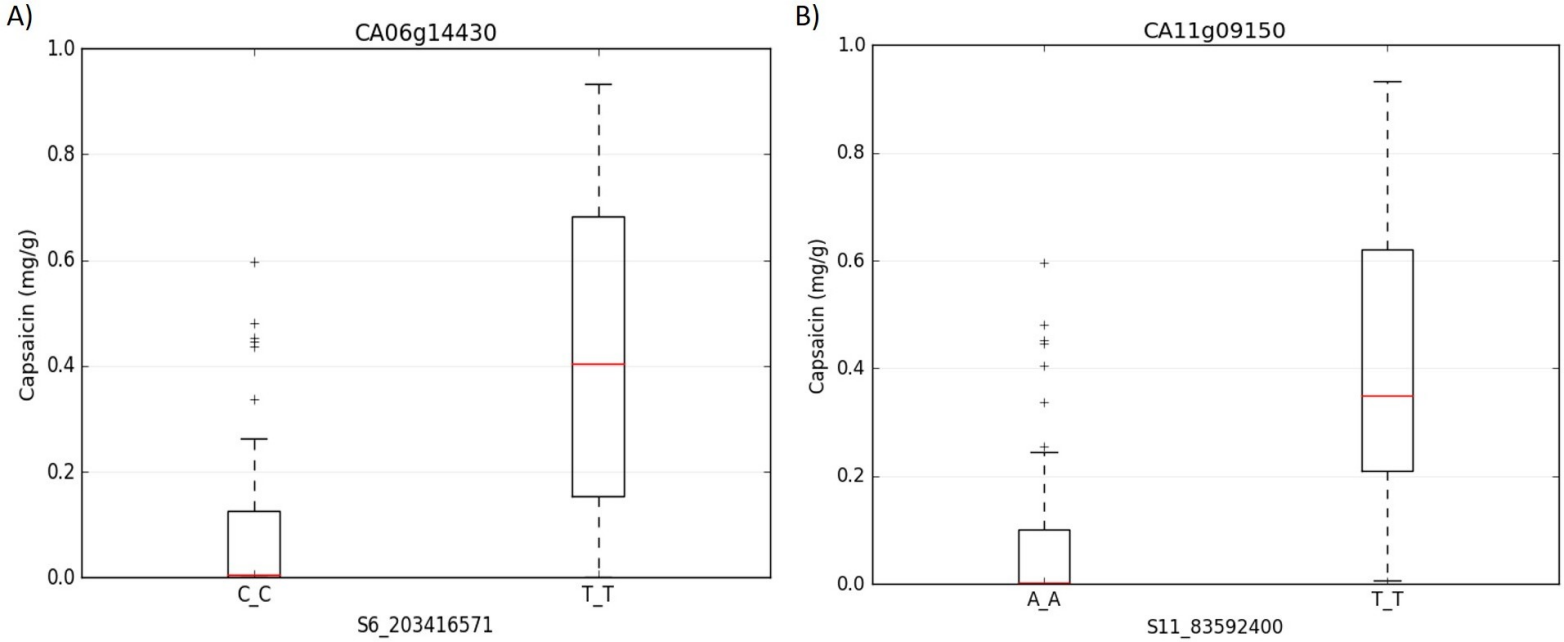
Allelic effect of two significantly associated SNPs markers for capsaicin content in *C*. *annuum*. Each plot is labeled with the SNP position in the X-axis. Y-axis represents the values for capsaicin levels (mg·g^-1^) in pepper powder from dried green fruit. Boxplot A) shows the effect of SNP marker in locus CA06g14430 on chromosome 06, whereas boxplot B) shows the effect of SNP marker in locus CA11g09150 on chromosome 11.

Capsaicinoids biosynthesis is carried out principally in the placental tissues of pepper fruits by the action of several enzymes [55, 56]. Recently, NGS approaches including genotyping by sequencing (GBS), based GWAS and RNAseq analysis of placenta tissues have been used for the identification of novel genes involved in the capsaicinoids biosynthesis pathway. Moreover, these approaches have allowed the study of the mechanisms involved in the pungency modulations in pepper. Liu et al. [57] predicted the function of three novel genes i.e., dihydroxyacid dehydratase (DHAD), threonine deaminase (TD) and prephenate aminotransferase (PAT) which play key roles in the capsaicinoids biosynthetic pathway. In a recent association mapping study carried out by Nimmakayala et al. [5], it was identified significant SNPs associated with capsaicin content and fruit weight. This study revealed that genes such as Ankyrin-like protein, IKI3 family protein, pentatricopeptide repeat protein and ABC transporter G and C subfamilies are important players regulating capsaicin content. The SNPs associated with the ABC transporter gene family were S6_203416571 and S11_83592400 in the locus CA06g14430 and CA11g09150 respectively (Fig 2). Particularly, the SNP S6_203416571 located in chromosome 6, showed a high allelic effect (Fig 2A).

### Genome-wide identification of ABC proteins in pepper

**Fig 3:**
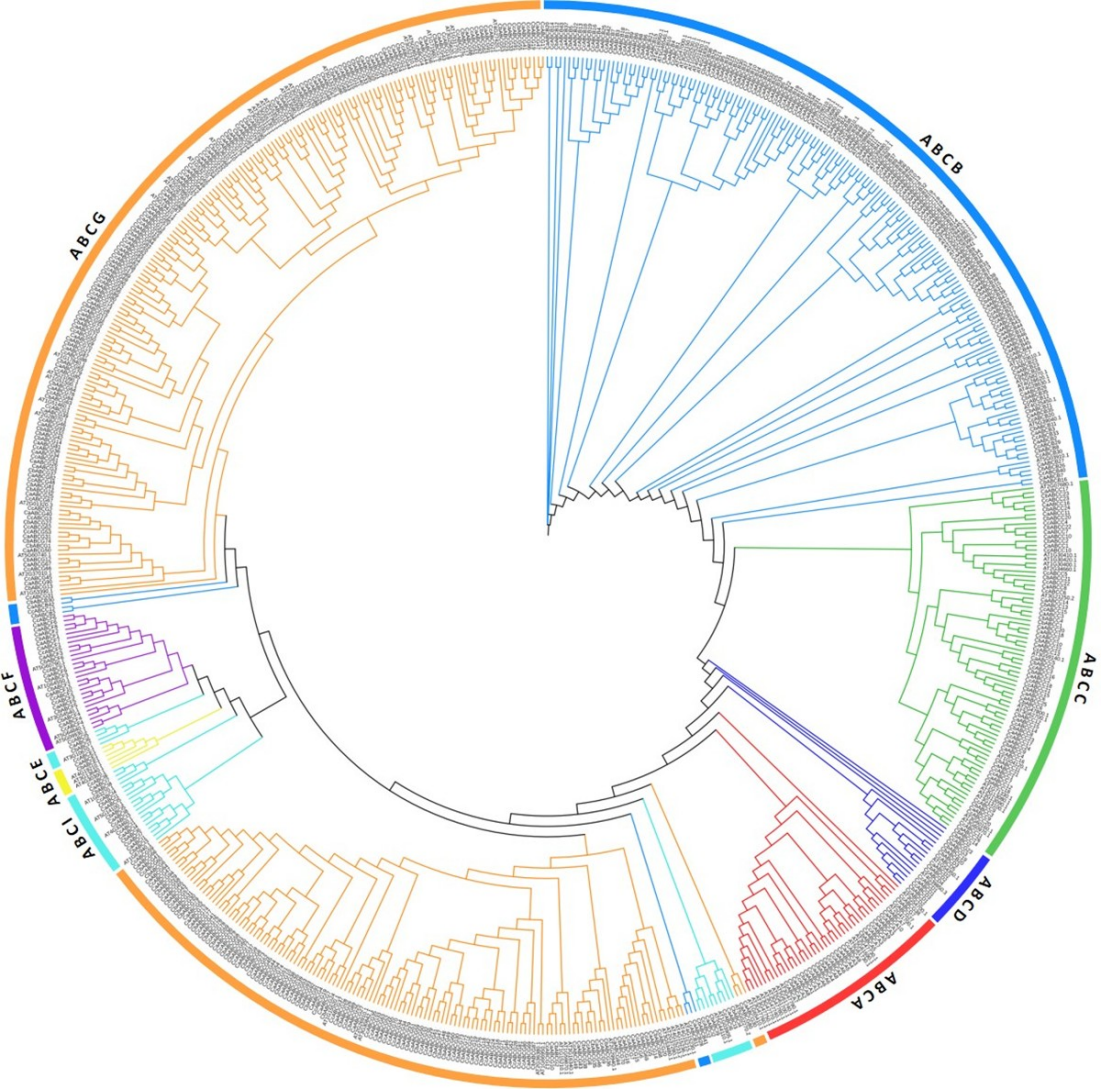
Phylogenetic relationships of *Capsicum* species and *Arabidopsis* ABC transporter proteins. The 572 and 119 ABC proteins identified from Capsicum species and Arabidopsis, respectively, were subjected to phylogenetic analysis by the neighbor-joining method with 1000 bootstrap replicates. Subfamily names (ABCA-I, except ABCH) are indicated by different colors.

To identify the ABC protein family in pepper, we performed a BLASTP search of the three pepper genomes from the PGP database. A total of 572 genes potentially encoding ABC proteins were identified: 200 from *C*. *annuum* (CaABC), 185 from *C*. *baccatum* (CbABC), and 187 from *C*. *chinense* (CcABC) (Table 1). To investigate the evolutionary relationship between *Capsicum* species and *Arabidopsis* ABC transporter proteins (AtABC), we performed phylogenetic analysis of the pepper and *Arabidopsis* ABC proteins. The protein sequences of *Capsicum* ABC genes and AtABC proteins (119 protein sequences containing the ABC transporter domain) were aligned by using MEGAX, and an unrooted phylogenetic tree was constructed by a NJ method with 1000 bootstrap replications (Fig 3).

**Table 1:** Comparative analysis of ABC proteins between Capsicum and other plant species.

Species name	ABC transporter subfamilies								Total ABCs transporter	Reference
	ABCA	ABCB	ABCC	ABCD	ABCE	ABCF	ABCG	ABCI		
*Arabidopsis thaliana*	12	29	15	2	3	5	43	21	130	[13]
*Arabidopsis lyrata*	12	25	15	2	3	10	43	22	132	[20]
*Brassica rapa*	11	38	21	2	7	7	63	30	179	
*Populus trichocarpa*	6	40	29	3	2	4	78	42	204	
*Glycine max*	8	49	40	10	2	10	113	39	271	
*Carica papaya*	5	18	13	3	2	4	36	32	113	
*Vitis vinifera*	5	30	26	1	1	6	71	41	181	
*Brachypodium distachyon*	6	32	19	4	4	7	44	22	138	
*Amborella trichopoda*	4	19	17	2	2	5	46	42	137	
*Oryza sativa*	6	28	17	3	1	6	56	24	141	
*Oryza sativa*	6	27	17	3	2	6	50	16	127	[16]
*Zea mays*	6	31	13	4	2	7	54	13	130	[18]
*Brassica napus*	30	69	47	5	13	14	116	20	314	[21]
*Ananas comosus*	5	20	16	2	1	5	42	9	100	[58]
*Solanum lycopersicum*	9	29	26	2	2	6	70	10	154	[59]
*Capsicum annuum*	10	48	26	2	1	10	95	8	200	
*Capsicum baccatum*	7	41	24	3	1	6	94	9	185	
*Capsicum chinense*	9	44	23	5	1	6	91	8	187	

An extensive research on ABC transporters has resulted in several naming schemes. In most of the cases, the transporters were named on the basis of mutant characteristics. Thus, different names were assigned to the same subfamily or selected members with common characteristics. To conform to plant and animal ABC communities, the Human Genome Organization (HUGO) nomenclature system [10] was adopted to designate all putative ABC proteins as ABCA-G and ABCI to all ABC transporter subfamilies. Overall, *Capsicum* ABC proteins followed the same pattern as *Arabidopsis* (Fig 3). Based on phylogenetic association with AtABCs and using the jackHmmer tool, *Capsicum* ABCs were classified into eight subfamilies previously mentioned. The number of members of ABCs within each subfamily in *Capsicum* were similar to other plants such as *Arabidopsis* [13], *B*. *rapa* [20] and tomato [59]. In order of abundance, ABCG, ABCB and ABCC subfamilies were the most prevalent groups throughout all species, whereas the smallest number of members were in the ABCD and ABCE subfamilies; for this last subfamily, only one member was identified in all the three *Capsicum* species analyzed.

For convenience, the ABC transporters were named CaABC1 to CaABCn for *C*. *annuum* based on their subfamily group and were classified similarly for the other species. The *Capsicum* ABC proteins vary substantially in size and sequences of their encoded region, as well as in their physicochemical properties across all species. The locations of the ABC domains within the protein also differ. The physical locations, coding sequence length, protein characteristics and topology for ABC transporters identified for each species are in S1–S3 Tables. The domain organizations for ABC transporters are almost as varied as their function: proteins of the ABCA-ABCD subfamilies have a forward direction for domain organization (TMD-NBD), whereas the proteins of the ABCG and ABCH subfamilies contain the reverse domain organization (NBD-TMD). ABCE and ABCF proteins contain only two NBDs and were characterized as soluble proteins. ABCI proteins generally possess only one domain, mainly NBD or TMD. Topological diversity is one of the unique characteristics of ABC proteins. The ABC transporters are divided in three common arrangements: full-sized transporters, half-sized transporters and a third type that has no TMDs but two NBD domains [10]. A typical full-sized ABC protein consists of ≥1,200 amino acid residues [14]. The 200 CaABC proteins ranged from 52 to 1831 amino acid residues, the CbABCs from 89 to 1864 residues and the CcABCs from 86 to 1965 residues. Nevertheless, it is important to mention that all of them possess at least one NBD, thus, they can be classified as ABC transporters and were included in this study. Some of the pepper ABC proteins with shorter sequences might be thought as pseudogenes or not annotated genes. These shorter sequences were also found in the genome-wide analysis of ABC transporters in tomato, *B*. *rapa* and pineapple [20, 58, 59]. Among the 572 ABC transporters, 212 lack a TMD and were considered soluble ABC proteins. The remaining 360 members possess TMDs and were considered ABC transporters across all species. Overall, 134 *Capsicum* ABC proteins are full-sized proteins possessing (TMD-NBD)x2 domains: 46, 40, 48 for *C*. *annuum*, *C*. *baccatum* and *C*. *chinense*, respectively. Among these members, 22, 24 and 28, respectively, exhibit a forward topology (TMD-NBD), whereas 24, 26, and 20 have a reverse topology (NBD-TMD). In total, 135 ABC transporters were classified as half-sized, having forward (TMD-NBD) or reverse (NBD-TMD) orientations. Among the half-sized *Capsicum* ABC proteins, 26 exhibit a forward and 109 a reverse domain orientation. A total of 233 ABC transporters were considered quarter-sized or single-structure proteins: 184 have an NBD domain, and 49 a TMD domain. *Capsicum* ABC proteins were also classified under an ABC2 (NBD-NBD) structure: 26 have the NBD-NBD structure and 3 the TMD-TMD structure. In total, 37 ABCs were uniquely characterized, with NBD-TMD-NBD, TMD-NBD-TMD and TMD-TMD-NBD-TMD-TMD structures. The differences in the topology domain orientations might have resulted from gene duplication during evolution or evolved to render specific physiological functions under biotic or abiotic stress [60].

### Chromosomal locations and syntenic *Capsicum* ABC paralog pairs

**Fig 4:**
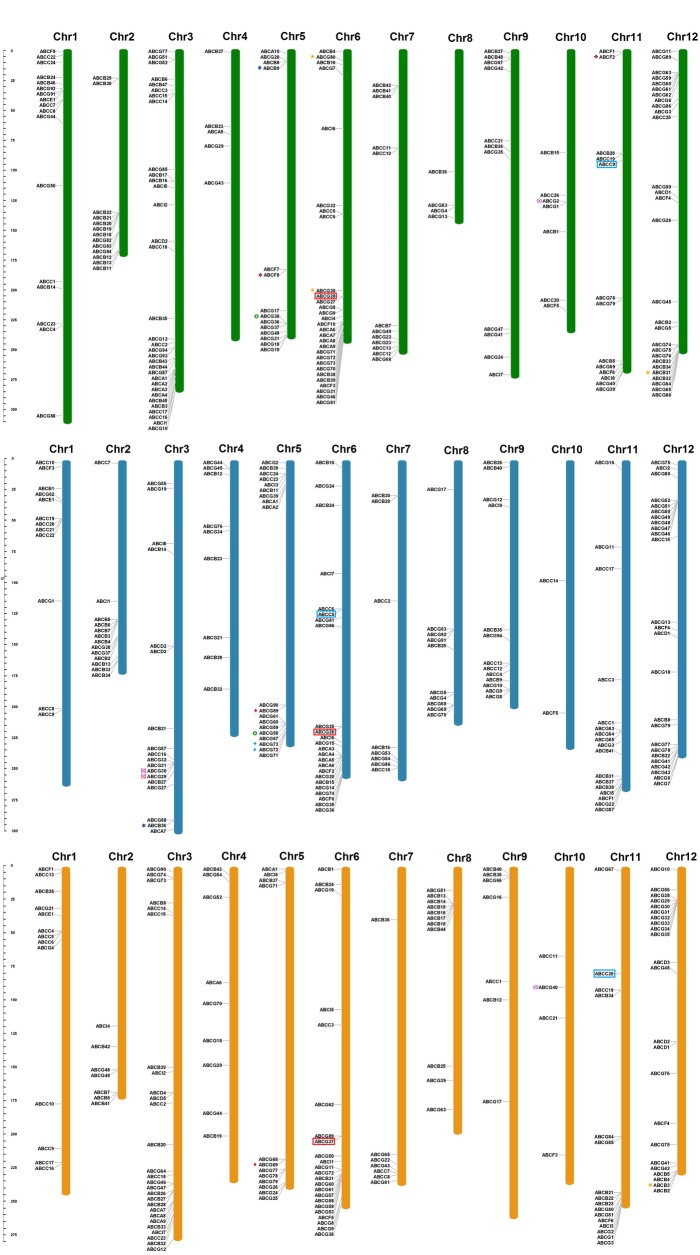
Chromosomal locations of ABC transporter proteins in pepper *C*. *annuum* (green), *C*. *baccatum* (blue) and *C*. *chinense* (orange). Chromosome numbers are represented at the top of each chromosome. The left panel scale indicates the chromosome length in Mb. The paralogous ABC gene pairs are represented with different colors and shapes. Orthologs genes of CA06g14430 and CA11g09150 are represented by red and blue boxes respectively.

A total of 544 (95.1%) ABC transporters were physically mapped on all 12 chromosomes of pepper, and the other 28 genes were located on unanchored scaffolds (Fig 4). ABCG, ABCB and ABCC subfamilies are unevenly distributed across all chromosomes. ABCD (in chromosome 2 and 12) and ABCE (in chromosome 01) subfamilies are the most conserved across all the *Capsicum* species. Among all chromosomes, chromosome 3 of *C*. *annuum* contains the highest number of ABCs—32 (16%)—followed by chromosome 6 (14.5%). Among all species, chromosomes 3, 6 and 12 contain the highest number of ABCs, with the minimum on chromosome 10.

The distribution pattern of ABC transporters on individual chromosomes also indicated certain physical regions with a relatively higher accumulation of multiple ABC gene clusters, such as chromosome 3 and 6 at the lower end of the arms for all species. The distribution of ABC transporters differs among the three genomes. Some ABC gene clusters occur in one species but not in the other genomes; for example, in chromosome 2, ABCs were present in the upper chromosome part in *C*. *annuum* and *C*. *baccatum* but were absent in *C*. *chinense*. On the other hand, ABCG and ABCB family members were found on at the lower part of chromosome 4 in *C*. *chinense* and *C*. *baccatum* but not in *C*. *annuum*. A clear example is at the upper end of chromosome 8, where a cluster of genes corresponding to the ABCB and ABCG members are present in *C*. *chinense*, and only one gene appears in *C*. *baccatum*, but with no presence reported in *C*. *annuum*.

Syntenic paralogs are genes that are located in syntenic fragments. The syntenic paralog pairs were identified between and within the three *Capsicum* genomes. Simultaneously, we further identified synonymous (Ks) and non-synonymous (Ka) values to explore the selective pressures on these paralog pairs to understand the expansion of this gene family in pepper. In total, 14 pairs of ABC transporter syntenic paralogs were identified across all species (Table 2). Six paralog pairs were within species and the remaining were intra-species. Among the eight intra-species duplications, three segmental duplication gene pairs were intra-chromosomal, located on chromosomes 5 and 3 for *C*. *baccatum* and chromosome 6 for *C*. *annuum*. Only one segmental duplication CaABCF8-CaABCF2 in *C*. *annuum* involved two different chromosomes. Moreover, the duplicated paralog ABC transporter pairs belong to the same subfamily. The Ka/Ks (ω) ratios for segmental duplications ranged from 0.06 to 1.57, with a mean of 0.81. In total, 11 out of 14 of the paralogs pair were under purifying selection, with ω ratios < 1. The ω ratios for 3 syntenic paralogs (21.42%) were >1, which indicates a positive selection on these paralogs. The CaABCF8-CaABCF2 pair had the highest ω ratios with 1.57.

**Table 2:** Ka-Ks calculation of each pair of syntenic Capsicum ABC paralogs.

Syntenic paralog pairs	S-Sites	N-Sites	Ka	Ks	Ka/Ks	Selection pressure	Duplication time (MYA)
CbABCG11-CbABCG40	127.23	406.77	0.23	0.21	1.06	Positive selection	15.36
CaABCG2-CcABCG40	142.31	490.69	0.23	0.25	0.89	Purifying selection	18.13
CaABCF8-CaABCF2	91.15	310.85	0.07	0.05	1.57	Positive selection	3.25
CcABCC5-CcABCC22	88.18	256.82	0.11	0.15	0.72	Purifying selection	11.12
CbABCB36-CaABCB9	260.28	843.72	0.00	0.02	0.06	Purifying selection	1.40
CaABCB31-CcABCB3	326.66	996.34	0.07	0.08	0.82	Purifying selection	6.00
CaABCG3-CaABCG34	49.75	166.25	0.10	0.09	1.13	Positive selection	6.11
CaABCG30-CaABCG80	108.39	371.61	0.52	0.54	0.95	Purifying selection	39.06
CbABCG89-CcABCG69	497.18	1704.82	0.02	0.08	0.31	Purifying selection	5.47
CaABCG23-CaABCG33	401.44	1314.56	0.01	0.01	0.47	Purifying selection	0.99
CbABCG7-CcABCG38	84.41	281.59	0.17	0.20	0.84	Purifying selection	14.57
CbABCG72-CbABCG73	52.13	184.87	0.82	1.00	0.82	Purifying selection	72.07
CbABCG29-CbABCG30	95.05	327.95	0.96	1.16	0.83	Purifying selection	83.55
CbABCG58-CaABCG38	148.66	514.34	1.01	1.15	0.88	Purifying selection	82.39

S-Sites, number of synonymous sites; N-Sites, number of non-synonymous sites; Ka, non-synonymous substitution rate; Ks, synonymous substitution; MYA, million years ago.

The duplication time of *Capsicum* ABC paralog pairs was estimated by using a relative Ks measure as a proxy for time, and it spanned from 1 to 84 million years ago (MYA), with an average duplication time of ~26 MYA. Multiple copies of genes in a gene family could have evolved due to the flexibility provided by events of whole-genome tandem and segmental duplications. Gene duplication, segmental or tandem, has been documented in several plant gene families, such as NAC, MYB, F-box, bZIP and ABC transporters [20, 61]. The ω ratios for 3 pairs of paralogs were > 1, representing positive selection and fast evolutionary rates in these ABC paralogs at the protein level. This finding differs from other gene families in plants, such as BURP in Medicago and ACD in tomato, which contain a few or even no paralog pairs undergoing positive selection [62, 63]. In our study, a relatively large percentage (~ 21%) of ABC paralogs pairs underwent positive selection. We assumed that these paralog gene pairs might have evolved in order to acquire new functions and adjust to their living environment. Expression correlation analysis of syntenic ABC paralog pairs across different tissues and under stress treatments could help to reveal their functional roles in evolutionary fates.

### Motif composition and *Cis*-elements of *Capsicum* ABC genes

**Fig 5:**
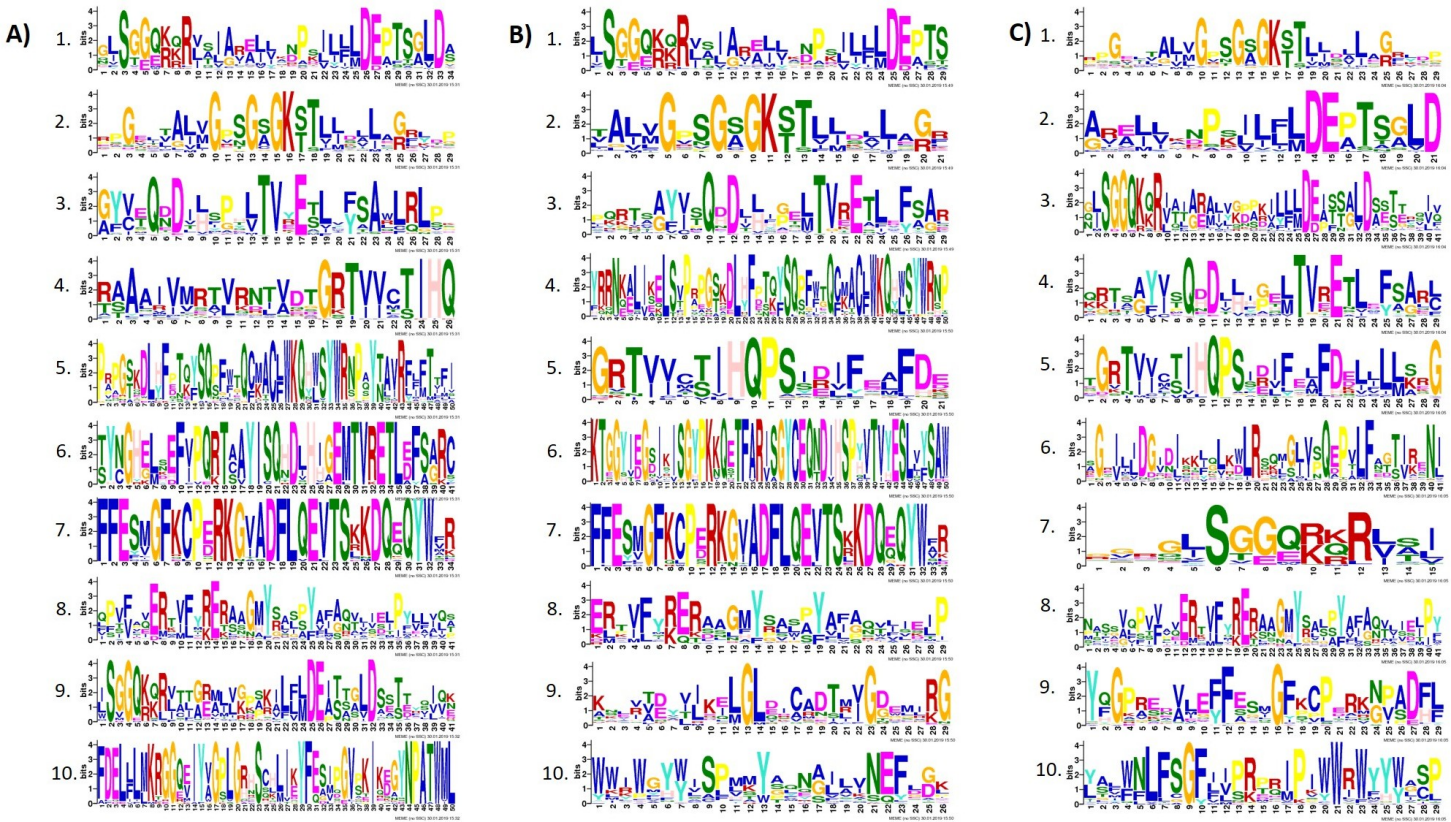
Conserved motifs of ABC transporter proteins in Capsicum species. (A) *C*. *annuum*, (B) *C*. *baccatum* and (C) *C*. *chinense*.

MEME analysis according to domain composition of pepper ABC transporter proteins revealed 10 conserved motifs in ABCA-G and ABCI families (Fig 5 and S4 Table). The lengths of the conserved motifs ranged from 15 to 50 amino acids. Additionally, the number of conserved motifs in each *Capsicum* ABC transporters ranged from 1 to 8. The information obtained from ScanProsite analysis revealed that the function of most of the motifs was pleiotropic drug resistance related to the ABCG subfamily. All conserved motifs predicted have similar properties as ABC transporters, and the signature motif (LSGGQ) was found in most of the *Capsicum* ABC transporters.

In order to identify putative *cis*-elements in the *Capsicum* ABC promoters, 1500 bp DNA sequences upstream of the start codon (ATG) for the ABC transporters for each species were analyzed by using the Plant *Cis*-acting Regulatory DNA Elements (PLACE) website. The analysis identified 124 different *cis*-elements in all *Capsicum* ABC transporters. A total of 23 common *cis*-regulatory elements were present across all the promoter regions of the ABC transporters and were highly conserved among all *Capsicum* species (Table 3).

**Table 3:** Common putative *cis*-elements identified in the promoter sequences of ABC proteins genes in Capsicum species.

*Cis*-element	Signal sequence	SITE	Expression patern
ABRELATERD1	ACGTG	S000414	ABRE, etiolation, erd
GCN4OSGLUB1	TGAGTCA	S000277	GluB-1, glutelin, endosperm, seed, storage protein, GCN4 motif
TATABOX4	TATATAA	S000111	TATA, sporamin, phaseolin
CATATGGMSAUR	CATATG	S000370	SAUR, NDE, auxin
ASF1MOTIFCAMV	TGACG	S000024	TGACG, root, leaf, CaMV, 35S, promoter, auxin, salicylic acid
NTBBF1ARROLB	ACTTTA	S000273	rolB, Dof, auxin, domain B, root, shoot, meristem, vascular
ARFAT	TGTCTC	S000270	auxin, AuxRE, ARF, ARF1, Aux/IAA, SAUR, NDE, GH3, D1, D4
CAATBOX1	CAAT	S000028	CAAT, legA, seed
CCAATBOX1	CCAAT	S000030	HSE (Heat shock element), CCAAT box
HEXMOTIFTAH3H4	ACGTCA	S000053	hexamer, HBP-1A, HBP-1B, histone H3, CaMV, 35S, NOS, HBP-1
T/GBOXATPIN2	AACGTG	S000458	T/G-box, JA, pin2, LAP, MYC, wounding
TATCCAYMOTIFOSRAMY3D	TATCCAY	S000256	GATA, amylase, sugar, repression
LTRE1HVBLT49	CCGAAA	S000250	low temperature, LTRE
GT1CONSENSUS	GRWAAW	S000198	GT-1, light, TATA, TFIIA, TBP, HR, SAR, TMV, leaf, shoot
INRNTPSADB	YTCANTYY	S000395	initiater, light-responsive transcription, TATA-less promoter
TATCCAOSAMY	TATCCA	S000403	alpha-amylase, MYB proteins, gibberellin, GA, sugar starvation
TGACGTVMAMY	TGACGT	S000377	alpha-Amylase, cotyledon, seed germination, seed
ABREATCONSENSUS	YACGTGGC	S000406	ABA, ABF, bZIP factors
BOXIIPCCHS	ACGTGGC	S000229	Box II, Box 2, CHS, chs, light regulation
LRENPCABE	ACGTGGCA	S000231	CAB, cab, cab-E, CABE, light, leaf, shoot
WRKY71OS	TGAC	S000447	WRKY, GA, MYB, W box, TGAC, PR proteins
LTRECOREATCOR15	CCGAC	S000153	low temperature, cold, LTRE, drought, ABA, cor15a, BN115, leaf
TBOXATGAPB	ACTTTG	S000383	GAPB, glyceraldehyde-3-phosphate dehydrogenase, light-activated

Four common cis-regulatory elements, CATATGGMSAUR, ASF1MOTIFCAMV, NTBBF1ARROLB and ARFAT, were found related to plant hormones including auxin, auxin response factor (ARF) and Small Auxin-Up RNAs (SAUR), which suggests that these plant hormones could affect the expression of *Capsicum* ABC transporters and can affect the plant growth and development. The WRKY71OS *cis*-regulatory element is responsive to stresses caused by pathogens. Out of the 23-common *cis*-regulatory elements, TBOXATGAPB, BOXIIPCCHS, INRNTPSADB, and GT1CONSENSUS are thought to be required for transcriptional regulation by light. Two common *cis*-elements, CCAATBOX1 and LTRECOREATCOR15 were identified to response to low temperature, cold, drought and heat shock, which suggests that *Capsicum* ABC transporters might be involved in response to abiotic stress.

### GO annotation of ABC transporter genes

**Fig 6:**
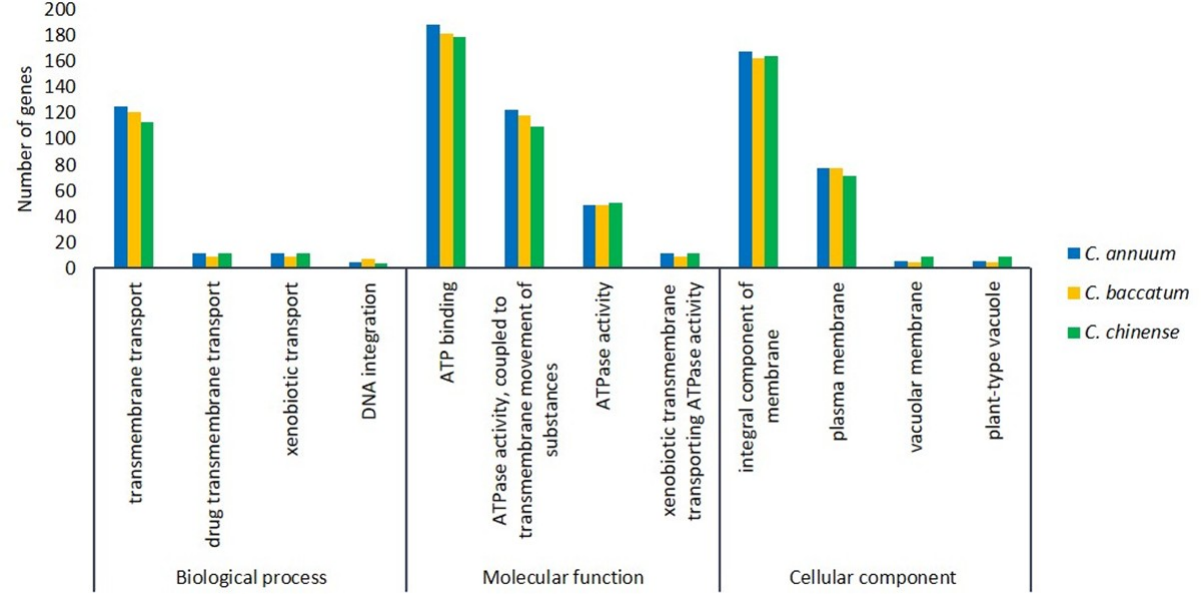
Detailed gene ontology analysis results for *Capsicum* species. Biological process, cellular component, and molecular function were identified with the Blast2GO program.

GO analysis performed with Blast2Go suggested the putative participation of ABC genes in multiple biological processes, molecular functions, and cellular component (Fig 6). GO results indicated the putative participation of *Capsicum* ABC transporters in transmembrane transport as a principal biological process, as well as drug transmembrane transport, xenobiotic transport and DNA integration. ATP binding and ATPase activity coupled to transmembrane of substances were the main activities for molecular function. Most of the ABC transporters were classified in the integral component of the membrane for cellular localization followed by the plasma membrane. In all species, 18 ABC transporters from *C*. *chinense*, 12 from *C*. *annuum* and 12 from *C*. *baccatum* were cellular localized in the vacuolar membrane and plant-type vacuole. In pepper fruit, capsaicinoids are synthesized exclusively in placental tissue and accumulate in vacuoles of placental epidermal cells [64], so ABC transporters might participate in vacuolar capsaicinoid uptake and transport, affecting the capsaicinoid content in pepper fruits.

Capsaicinoid levels are highly dynamic during fruit development. Their levels appear to be influenced by the ontogenetic trajectory of the fruit. Capsaicinoids begin to accumulate from the early stages (10 dpa) of fruit development, peak at about 40 dpa, and then it decreases sharply [65]. The late decrease in capsaicinoid content appears to result from high peroxidase activity, which oxidizes capsaicinoids in the presence of hydrogen peroxide (H_2_O_2_) [66, 67]. A gene CcABCC12 from *C*. *chinense* was found to have a H_2_O_2_ catabolic process as a biological process resulting in the breakdown of H_2_O_2_ (S5 Table), which suggests a detoxification process of H_2_O_2_ exclusively for *C*. *chinense* and a subsequent high content of capsaicinoids. Another factor that can affects the metabolism of capsaicinoids is mineral nutrition. Nitrogen (N) and potassium (K) are the main mineral players. Nitrogen availability in soil directly affects capsaicin accumulation since a single capsaicin molecule synthesis involves three amino acids such as phenylalanine, valine and leucine [68]. By contrast, potassium does not participate in capsaicinoid metabolism, however it has been reported that an increase in potassium concentration significantly decreases the capsaicin levels and leaf nitrogen content in *C*. *chinense* [69]. Thus, the level of potassium might indirectly affect capsaicin accumulation via its effects on fruit development [70]. CcABCC1 in *C*. *chinense* showed cellular potassium ion homeostasis as a biological process. The principal function of this biological process involves maintenance of an internal steady state of potassium ions at the level of a cell. In fact, *C*. *chinense* was found to have the highest values for capsaicin and dihydrocapsaicin, suggesting that cellular potassium ion homeostasis may indirectly affect capsaicinoid levels in pepper fruits.

### Expression profile of ABC transporters in *C*. *annuum* and *C*. *chinense*

**Fig 7:**
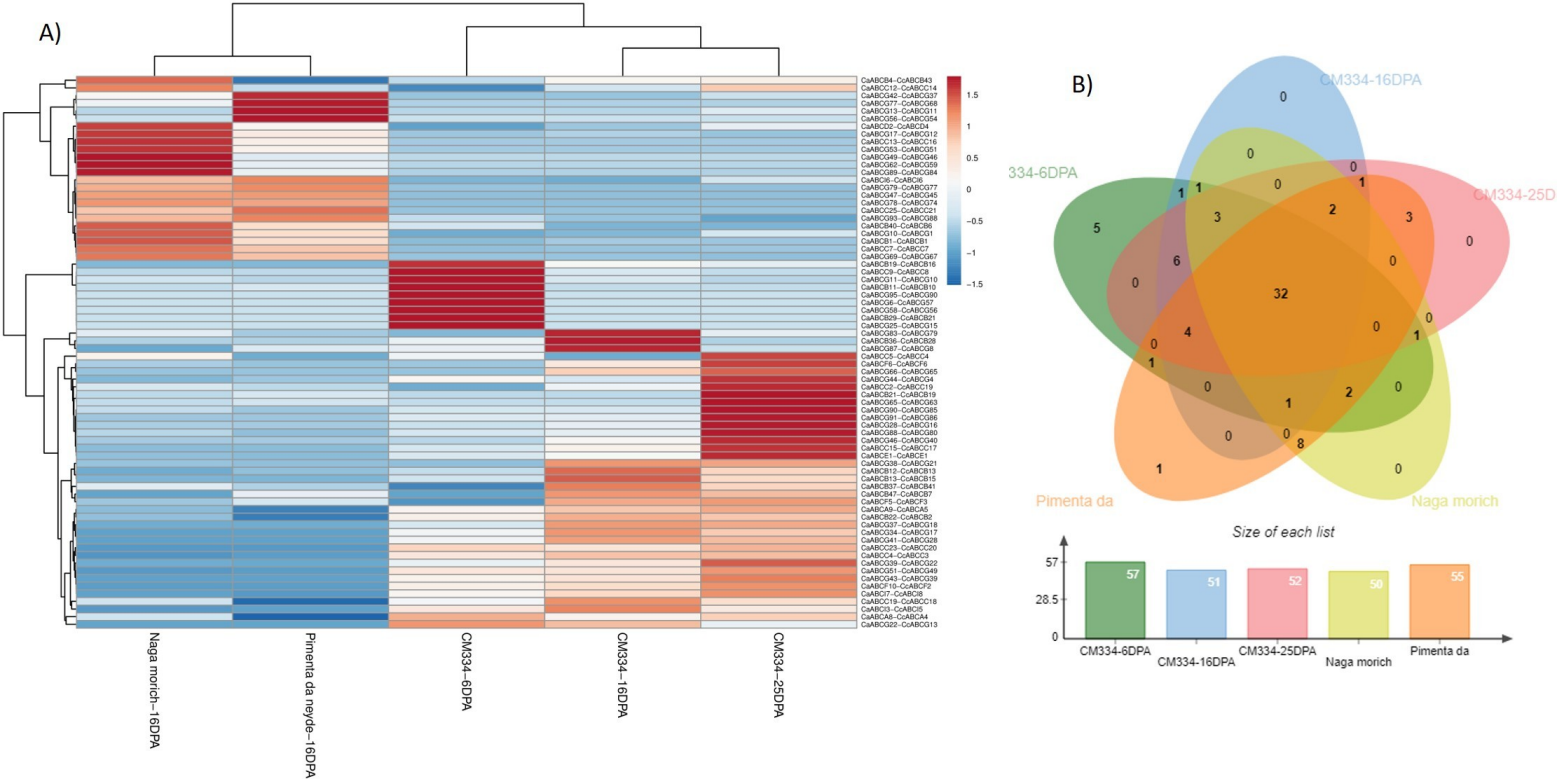
Expression patterns of ABCs transporters in placenta tissue of C. annuum var CM344 and C. chinense. A) Heat map of expression profiles (in log2-based RPKM) from placenta tissues (6, 16, 35 days post-anthesis (dpa)) of *C*. *annuum* and placenta tissues at 16 dpa of two *C*. *chinense* varieties (Naga morich and Pimienta da neyde). The expression levels are represented by the color bar: red, upregulated, and blue, downregulated. B) Venn diagram analysis of the tissue expression of CaABC (C. annuum) -CcABC (C. chinense) transporters.

A BLASTN strategy was used to identify the orthologs for Capsaicinoid markers previously identified by [5] for the CA06g14430 gene from the SGN database. The resulted orthologs were CaABCG28, CbABCG26, and CcABCG37 corresponding to each of the species. For CA11g09150, the orthologs were CaABCC9, CBABCC5 and CcABCC20. The main purpose of gene expression profiling is to determine the genes that are differentially expressed within the organism being studied. In the same way, we used a BLASTN search to identify the orthologs between *C*. *annuum* and *C*. *chinense* to correlate their expression in placental tissues. In order to characterize the expression patterns of individual *Capsicum* ABC transporters at different stages (6, 16 and 25 dpa), we used publicly available RNA-seq data for *C*. *annuum* cv. CM334 [2]. The RPKM values for green fruits (16 dpa) from two varieties of *C*. *chinense* (Naga morich and Pimienta de neyde) and *C*. *annuum* cv CM334 were plotted in a hierarchical heatmap (Fig 7).

The *C*. *annuum* CM334 variety showed a similar pattern of expression in all placenta tissue stages (Fig 7A). CaABCG11 was expressed at 6 and 16 dpa with a higher expression at 6 dpa. On the other hand, CaABCB36, CaABCG83 and CaABCG87 were highly expressed at 16 dpa. Most *Capsicum* ABC transporters presented different expression patterns, whereas a few resulted similar. Some exhibited stage- and species-specific expression, which suggests that these genes may play specific roles in the relevant stages and *Capsicum* species. Among 74 genes, 32 were expressed across all placenta tissues at different stages (Fig 7B). The ABC transporters previously described as a major marker for capsaicin and dihydrocapsaicin content were mostly expressed in *C*. *annuum* cv. CM334. CaABCC9 and CcABCC20 (CA11g01950) were found greatly expressed at 16 and 25 dpa and CaABCG28 (CA06g14430) was found in 25-dpa tissue. By contrast, CcABCG37 (CA06g14430) was greatly expressed only *C*. *chinense* cv. Naga morich at 16 dpa. Mainly, the ABCC and ABCG subfamilies were distributed across different stages; however, only ABCA, ABCB, ABCE, ABCF and ABCI members were expressed in *C*. *annuum* cv. CM334, and ABCD members were expressed in *C*. *chinense* cultivars. *C*. *chinense* varieties at 16 dpa shared the expression of eight genes (CcABCC16, CcABCC21, CcABCG45, CcABCG46, CcABCG51, CcABCG68, CcABCG74, CcABCG84). CcABCG54 was exclusively expressed in Pimienta de neyde, whereas CcABCG12, CcABCG16, CcABCG46, CcABCG51, CcABCG59, CcABCG84 and CcABCD4 were highly expressed in Naga morich. Most of the genes expressed in the *C*. *chinense* varieties belonged to the ABCC, ABCG and ABCD families.

The ABCA subfamily is not yet fully functionally characterized in plants; it has been reported to be related to pollen and seed germination and maturation [71]. The presence of one full-sized ABCA transporter was exclusive to dicots, including pepper, *Arabidopsis* [13], tomato [59], *B*. *rapa* [20], and *B*. *napus* [21] but so far it has not been identified in monocots, such as rice [16] and maize [18]. However, Chen et al. [58] reported one full-sized ABCA transporter in pineapple. The ABCB subfamily is composed of a full-sized or multidrug resistance (MDR) protein and half-sized protein, with names such as transporters associated with antigen processing (TAP) and ABC transporter of mitochondria (ATM) [10]. In plants, ABCB is the second largest subfamily. For instance, in *Arabidopsis*, the ABCB subfamily participates in different processes such as auxin bidirectional transport, phospholipid translocation, stomatal regulation, berberine transport, Fe/S biogenesis and metal stress (Cd and Al) tolerance [72]. AtABCB1, a member of AtABCB, has been proposed to participate in auxin transportation, and AtABCB1-overexpressing plants show long hypocotyls [73, 74].

ABCE family members are soluble ABC proteins and are also called RNase L inhibitor (RLI). They possess an N-terminal Fe-S domain, which interacts with nucleic acids [75]. Their main function have been reported to be related to control of translation and ribosome biogenesis [76]. Similarly, ABCE and ABCF family members are soluble proteins and contain an NBD-NBD domain structure. In *Arabidopsis*, ABCF (AtABCF3) proteins have been reported to play a role in root growth [77].

The ABCG subfamily, also called pleiotropic drug resistance or white–brown complex proteins, is the largest subfamily in plants. It has been reported that ABCGs transport various phytohormones, including abscisic acid, cytokinin, strigolactone and auxin derivatives [78]. The subcellular localization of full-sized ABCGs is the plasma membrane [79], whereas half-sized ABCGs are complex proteins and have been localized in the plasma membrane, mitochondrial membrane, chloroplast membrane and cytoplasm [18]. Full-sized ABCGs of *Arabidopsis*, AtABCG32 [80], and rice OsABCG31 [81] are involved principally in cuticle formation, while half-sized ABCGs play an important physiological role like cuticle formation, kanamycin resistance, abscisic acid exporter and pollen development [82–84]. In cotton, GhWBC1 a half-sized white-brown complex member has been reported to be involved in fiber cell elongation [85]. Shibata et al. [86] reported that ABCG subfamily may play a key role in export of the antimicrobial diterpene such as sesquiterpenoid phytoalexin and capsidiol for resistance to the potato late blight pathogen *Phytophthora infestans* in *Nicotiana benthamiana*.

The ABCC subfamily is also called MDR-associated proteins (MRP) because of their function in transporting glutathione- and glucuronide-conjugates in drug resistance (Verrier et al., 2008). Pang et al. [18] reported that most plant ABCCs are characterized as vacuolar localized proteins and a few of them have been reported to reside on the plasma membrane. The function of different ABCC members has been found in diverse plants; for example, *Arabidopsis* AtABCC5 [87], maize ZmMRP4 [88] and rice OsABCC13 [89] are implicated in phytate transport. AtABCC1 and AtABCC4 are involved in folate transport, while maize ZmMRP3 and grape VvABCC1 play an important role in anthocyanin accumulation in vacuoles [90, 91]. The high expression of the ABCC subfamily in pepper placental tissue of two species, and its principal function reported in other plants, suggest that the ABCC subfamily function in *Capsicum* spp. is the transport and accumulation of capsaicin in vacuoles of the placental tissue.

### Gene expression analysis

**Fig 8:**
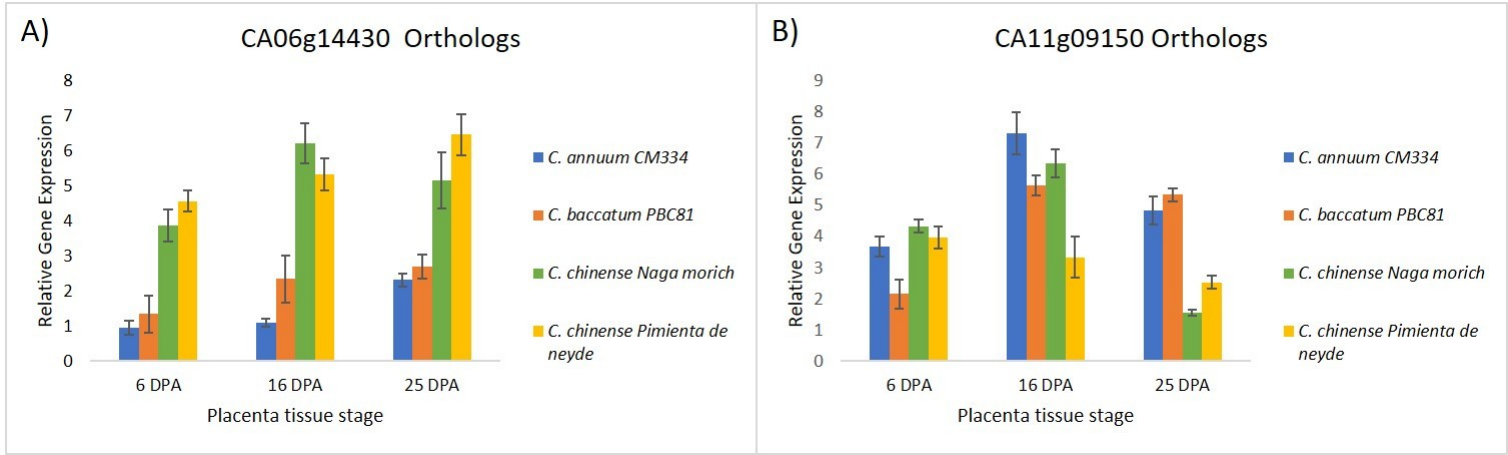
Gene expression analysis of selected ABC transporters in placenta tissues at 6, 16 and 25 days post-anthesis (dpa) across Capsicum species. RT-qPCR analysis for A) CA06g14430 homologs (CaABCG28, CbABCG26, and CcABCG37) and B) CA11g09150 orthologs (CaABCC9, CBABCC5 and CcABCC20). Values are means ± SD; n = 3.

We selected CaABCG28, CbABCG26 and CcABCG37 (orthologs of CA06g14430); as well as CaABCC9, CBABCC5 and CcABCC20 (orthologs of CA11g09150) for gene expression analysis by RT-qPCR (Fig 8). Gene expression was detected throughout all placenta stages and species analyzed. The orthologs of CA06g14430 corresponding to the ABCG family showed a similar expression pattern in the *C*. *chinense* varieties at different stages, but at 16 dpa, higher relative expression was found for the Naga morich variety and the lowest was for *C*. *annuum* cv. CM334 (Fig 8A). At 6 dpa, the expression was similar across all cultivars, but it was less in *C*. *baccatum* and *C*. *chinense* cv. Pimienta de neyde at 16 dpa. The remaining varieties showed an expression pattern close to that of the ABCC family (Fig 8B). At 25 dpa, the highest expression was found for *C*. *annuum* cv. CM334, followed by *C*. *baccatum* cv. PBC81 and the lowest for the *C*. *chinense* varieties. Different patterns in the expression between the orthologs of the Capsaicinoids markers previously mentioned suggest that the expression may be specie-specific for each of the ABC subfamilies in *Capsicum* species.

## Conclusion

Although the ABC transporter gene superfamily has been widely studied among extant organisms including plants, the present study is the first to report the presence of 572 putative ABC transporter proteins in the entire pepper genome sequences of three different *Capsicum* species. Our results provide fundamental and exhaustive information about the pepper ABC transporters by performing a comprehensive genome-wide identification and expression patterns of these proteins family. Based on their evolutionary origin, phylogenetic analysis classified the ABC proteins into 8 main subfamilies (designated A to G, and I). Chromosomal mapping revealed that members of ABCG, ABCB and ABCC subfamilies were the most abundant genes, whereas the ABCD and ABCE subfamilies were manifested in a lesser abundance. Our results suggest that the ABC transporters, specifically the ABCC and ABCG subfamilies, interfere in capsaicin and dihydrocapsaicin content in pepper. Indirectly, these two subfamilies may be involved in the transportation of secondary metabolites such as capsaicinoids to the placenta vacuoles for their storage. Moreover, we suggest that the ABBC and ABCG subfamilies play a role in the H_2_O_2_ detoxification process to reduce capsaicin degradation, specifically in the *C*. *chinense* fruits. Our study will provide clues for further research on the evolution of the ABC transporter gene family and their influence in specific biological functions of *Capsicum* fruits including plant growth, development and capsaicinoid content in pepper.

## Supporting information

S1 Table Basic information on the ABC transporter gene family in *C. annuum*. (XLSX)

S2 Table Basic information on the ABC transporter gene family in *C. baccatum*. (XLSX)

S3 Table Basic information on the ABC transporter gene family in *C. chinense*. (XLSX)

S4 Table Conserved motifs present in ABC transporter in *Capsicum* species. (XLSX)

S5 Table Detailed information on gene ontology annotation results for *Capsicum* species. (XLSX)

S6 Table RPKM values for placenta tissues of orthologs of ABC transporter pairs of *C. annuum* and *C. chinense*. (XLSX)
